# IOL tilt and decentration: a comparison of different haptic designs using CNN and SS-OCT in a short term

**DOI:** 10.3389/fmed.2026.1750166

**Published:** 2026-01-22

**Authors:** Jinhan Yao, Kaiwen Li, Feiyan Chai, Jingjing Wang, Zhao Wang, Xiaogang Wang

**Affiliations:** 1 Shanxi Medical University, Taiyuan, China; 2 Institute of Medical Technology, Peking University Health Science Center, Peking University, Beijing, China; 3 Shanxi Eye Hospital Affiliated to Shanxi Medical University, Taiyuan, China; 4 School of Electronic Science and Engineering, University of Electronic Science and Technology of China, Chengdu, China

**Keywords:** cataract surgery, decentration, haptic, intraocular lens, tilt

## Abstract

**Purpose:**

To investigate the tilt and decentration differences and influencing factors of intraocular lenses (IOLs) with plate-haptic and C-loop haptic designs one month after cataract surgery, using the combination of swept-source optical coherence tomography (SS-OCT) and a customized convolutional neural network (CNN) algorithm.

**Methods:**

Participants were categorized into two groups based on the haptic design of their implanted IOLs. Group A included 37 patients (37 eyes) with the ATTORBI 709 M plate-haptic design, while Group B comprised 42 patients (42 eyes) with the PY-60 AD C-loop design. SS-OCT examinations were performed both before and one month after cataract surgery. A customized CNN algorithm was developed to generate data for IOL tilt and decentration, using the corneal vertex as a reference.

**Results:**

This study included 79 patients (79 eyes). Before surgery, there were no statistically significant differences in ocular parameters between the two groups (all *p* > 0.05). Significant difference in IOL decentration was observed (Group A & Group B: 0.16 ± 0.08 mm & 0.42 ± 0.28 mm, *p* < 0.001). In contrast, the difference in IOL tilt was not significant (Group A & Group B: 4.60 ± 1.27° & 4.63 ± 1.93°, *p* = 0.948). Both groups displayed a predominant inferotemporal tilt of the crystalline lens and IOL, but decentration did not show a distinct distribution pattern. Multivariate linear regression confirmed a correlation between IOL tilt in both groups and crystalline lens tilt (*p* < 0.003).

**Conclusion:**

The plate-haptic design IOL shows less decentration than the C-loop design. Clinicians should consider this potential difference in IOL stability when selecting IOLs with varying haptic designs.

## Introduction

The decentration and tilt of intraocular lenses (IOLs) following cataract surgery are critical determinants of patients’ visual quality. Research indicates that when IOL tilt exceeds 7° or decentration surpasses 0.4 mm, there is a notable increase in postoperative wavefront aberrations and a decrease in the capability of cylinder correction, especially for eyes with premium multifocal, Toric, and extended depth of focus (EDOF) IOL implantations in different corneal conditions (1–5). Variations in IOL haptic designs, such as C-loop and plate-haptic configurations, as well as differences in overall size, may influence the IOL’s orientation (6, 7). Clinically, ultrasound biomicroscopy (UBM) can be employed to analyze crystalline lens and IOL positioning, though it is a contact-based procedure that requires the patient to be supine. The supine position and the lack of a stable fixation target may affect measurement reliability (8). Purkinje imaging can assess the position of the crystalline lens or IOL by detecting light reflections from different refractive surfaces. Moreover, its reliance on the IOL’s curvature radii for calculations can introduce errors, particularly with relatively flat IOLs (8, 9). Furthermore, the potential shift in the pupil center with varying pupil sizes suggests that using the pupil axis as a reference can lead to fluctuations in tilt and decentration measurements, thereby reducing their repeatability (10–12).

Pentacam Scheimpflug imaging and swept-source optical coherence tomography (SS-OCT) enable rapid anterior segment imaging, facilitating the measurement of tilt and decentration metrics. However, the Pentacam requires a minimum pupil diameter of 6 mm to detect the crystalline lens posterior surface accurately, and fluctuations in the pupil axis can also affect measurement reliability (10, 11, 13, 14). Some commercial SS-OCT devices automatically provide crystalline lens or IOL tilt and decentration values via integrated software (2, 15, 16). Yet, the lack of a universally accepted analytical algorithm leads to variability in the determination of these metrics across different imaging qualities (9). Our research on IOL tilt and decentration analysis has progressed significantly over the past decade. In 2013, we conducted initial analyses using time-domain OCT, referencing the pupillary plane (17). By 2021, we had advanced to using SS-OCT images and the corneal vertex as a reference axis, achieving repeatable measurements of IOL tilt and decentration (18). While these earlier methods provided reliable results, they were not fully automated. To enhance analysis efficiency, our team published a robust spatial-position analysis algorithm in 2023. This algorithm, designed for both crystalline lenses and IOLs, is based on multitask learning and was developed using a dataset of 1,251 SS-OCT images from 180 patients. It demonstrated excellent inter-observer performance for segmentation and calculation, with Dice coefficients higher than 0.968 (19). In the current study, we apply this same algorithm to evaluate IOL tilt and decentration, continuing to use the corneal vertex as a dependable reference (18).

The aims of various IOL haptic designs and their subsequent modifications are twofold: first, to enhance the IOL’s postoperative stability, and second, to reduce the frequency of posterior capsule opacification. These haptic configurations predominantly encompass the open-loop plate haptic design (such as the Akreos Adapt), the whole plate haptic design (such as Zeiss 509 M), the single-piece C-loop design (such as AcrySof SA60AT and Lenstec SOFTEC HD), the open-loop single-piece C-loop design (such as Rayner 920H), and the three-piece C-loop design (such as AcrySof MA60AC) (11). However, the existing literature indicates a scarcity of direct comparative analyses between the three-piece C-loop design (PY-60 AD) and the whole-plate haptic design (Zeiss 709 M). The PY-60 AD is an aspheric, three-piece, hydrophobic acrylic IOL with modified C-loop haptics, a 5-degree angulation, and a contact length of 12.5 mm. In contrast, the Zeiss 709 M is an aspheric, single-piece, hydrophilic acrylic plate haptic IOL, characterized by its lack of haptic angulation and a 12.53 mm contact length (calculated with Pythagorean theorem).

This study aims to conduct an analytical comparison of the short-term postoperative spatial positioning of the PY-60 AD and 709 M IOLs. Furthermore, the study seeks to elucidate the pertinent factors influencing the postoperative positioning of these IOLs.

## Materials and methods

This cross-sectional, retrospective, observational study recruited 79 patients diagnosed with senile cataracts who underwent phacoemulsification and IOL implantation at the Shanxi Eye Hospital from January 2021 to May 2024. Patients were categorized into Group A (implanted with a plate-haptic design, AT TORBI 709 M, Carl Zeiss) and Group B (implanted with a C-loop design, PY-60 AD, HOYA, Japan). The study conformed to the Declaration of Helsinki, receiving approval from the Ethics Committee of Shanxi Eye Hospital Affiliated to Shanxi Medical University (No. 2019LL130), and all participants provided informed consent.

Participants eligible for this study included: senile cataract, 21.0 mm ≤ axial length (AL) ≤ 26.0 mm, and postoperative best-corrected visual acuity (BCVA) ≥ 12/20. Exclusion criteria encompassed coexisting ocular diseases (such as corneal pathologies, lens dislocation, or retinal disorders), systemic ailments that might influence ocular health (such as diabetes, hypertension, or hyperthyroidism), and intraoperative complications (including posterior capsule rupture, incomplete anterior capsulorhexis, or sulcus implantation of IOL), as well as severe preoperative refractive media opacities that hindered data collection.

For briefing the surgical procedure, following topical anesthesia, a 2.2 mm main clear cornea incision was created in the superonasal quadrant for the left eye or the superotemporal quadrant for the right eye. Subsequently, an ophthalmic viscosurgical device (OVD) was injected into the anterior chamber, and a 5.0–5.5 mm diameter continuous curvilinear capsulorhexis was manually created. The nucleus and cortical material were efficiently emulsified and aspirated. IOL was implanted, and the clear corneal incision was hydrated.

All enrolled patients underwent anterior segment SS-OCT imaging system (ANTERION; software version 1.3.4.0; Heidelberg Engineering, Heidelberg, Germany) prior to and one month after the cataract surgery. ANTERION uses a 1,300-nm light source to obtain ocular biometric parameters, including AL, anterior chamber depth (ACD), central corneal thickness (CCT), and lens thickness (LT), as well as anterior segment cross-sectional images.

Statistical analyses were conducted using SPSS software (version 22.0; SPSS, Chicago, IL, USA). The Shapiro–Wilk test was employed to assess the normality of the variables. For data following a normal distribution, an independent samples *t*-test was used. Otherwise, the Mann–Whitney U test was applied. Univariate and multivariate linear regression analyses were employed to evaluate the correlation of IOL position and preoperative biometric values, including AL, ACD, and LT, as well as the tilt and decentration of the crystalline lens. Model collinearity was assessed using variance inflation factors (VIFs). Statistical significance was determined at *p* < 0.05.

The sample size was calculated using MedCalc software (version 20.216; MedCalc Software Ltd). With a Type I error rate of 0.05 and a Type II error rate of 0.10, sample size calculations were performed. Based on a previous study, a clinically significant mean tilt difference of 1.32 degrees, with standard deviations of 0.89 and 1.83 degrees for Group 1 and Group 2, respectively, required a minimum of 27 eyes per group with a 1:1 ratio. Additionally, for a mean decentration difference of 0.1 mm and standard deviations of 0.13 mm and 0.06 mm for Group 1 and Group 2, a minimum of 23 eyes per group was needed with a 1:1 ratio (11).

## Results

A total of 79 patients (79 eyes) were included in this study. Table 1 shows no statistically significant differences in preoperative ocular biometrics, postoperative BCVA, and astigmatism magnitude between the two groups (all *p* > 0.05).

**Table 1:** Baseline characteristics of the two groups.

Parameters	Group A (37 eyes)	Group B (42eyes)	*p*
Age (years)	67.24 ± 11.17	69.57 ± 9.55	0.321
Male/Female	16/21	19/23	0.859
AL (mm)	23.47 ± 0.87	23.10 ± 1.02	0.094
CCT (μm)	528 ± 31	527 ± 27	0.957
ACD (mm)	3.26 ± 0.41	3.15 ± 0.38	0.231
LT (mm)	4.41 ± 0.45	4.44 ± 0.38	0.695
WTW (mm)	11.30 ± 0.46	11.27 ± 0.51	0.790
K flat (D)	43.76 ± 1.70	44.42 ± 1.55	0.076
K steep (D)	45.72 ± 1.62	45.23 ± 1.63	0.178
Crystalline lens tilt (°)	3.50 ± 1.00	3.33 ± 1.15	0.473
Crystalline lens decentration (mm)	0.16 ± 0.11	0.20 ± 0.14	0.152
Postoperative BCVA (decimal)	0.88 ± 0.30	0.85 ± 0.30	0.703
Postoperative astigmatism magnitude (D)	−0.21 ± 0.52	−0.30 ± 0.89	0.626

AL, axial length; ACD, anterior chamber depth; BCVA, best corrected visual acuity; CCT, central corneal thickness; D, diopter; K, keratometry; IOL, intraocular lens; LT, lens thickness; WTW, white to white.

**Figure 1:**
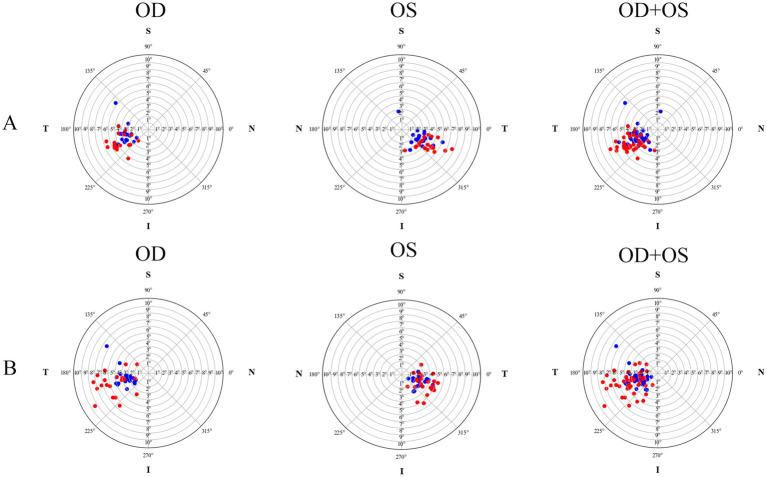
The tilt distribution pattern of crystalline lenses and intraocular lenses for both the plate-haptic group **(A)** and the C-loop group **(B)**. The blue markers indicate crystalline lenses, while the red markers represent the IOLs. OD refers to the right eye, and OS denotes the left eye.

**Figure 2:**
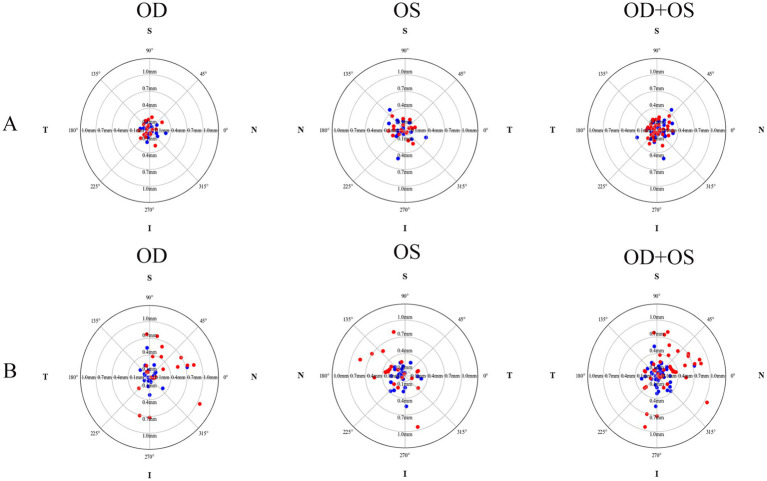
The decentration distribution pattern of crystalline lenses and intraocular lenses for both the plate-haptic group **(A)** and the C-loop group **(B)**. The blue markers indicate the crystalline lenses, while the red markers represent the IOLs. OD refers to the right eye, and OS denotes the left eye.

The postoperative IOL tilt in Group A and Group B was (4.60 ± 1.27)° and (4.63 ± 1.93)°, respectively, with no statistically significant difference (*p* = 0.948, Cohen’s d = 0.02). However, the difference in postoperative IOL decentration between Group A (0.16 ± 0.08 mm) and Group B (0.42 ± 0.28 mm) was statistically significant (*p* < 0.001, Cohen’s d = 1.26). As shown in Figure 1, the crystalline lens and the IOL in the two groups tended to incline toward the inferotemporal quadrant, whereas the decentration did not exhibit an obviously distinct distribution pattern (Figure 2). In Group A, 89.19% (33/37) of the crystalline lenses and 94.59% (35/37) of the IOLs inclined toward the inferotemporal quadrant with a tilt degree of less than 7°, and 91.89% (34/37) of the crystalline lenses had a decentration of less than 0.4 mm. All the IOL decentrations were less than 0.4 mm. In Group B, 95.24% (40/42) of the crystalline lenses and 69.05% (29/42) of the IOLs inclined toward the inferotemporal quadrant with a tilt degree of less than 7°, with 92.86% (39/42) of the crystalline lenses and 57.14% (24/42) of the IOLs with decentration of less than 0.4 mm.

In Group A, both univariate (*β* = 0.767, *p* < 0.001) and multivariate (*β* = 0.738, *p* < 0.001) linear regression analyses demonstrated a significant correlation between IOL tilt and preoperative crystalline lens tilt. Similarly, in Group B, both univariate (*β* = 0.774, *p* = 0.002) and multivariate (*β* = 0.759, *p* = 0.003) linear regression analyses confirmed that IOL tilt was exclusively correlated with preoperative lens tilt (Table 2).

**Table 2:** Univariate and multivariate linear regression analyses of IOL tilt with preoperative parameters in both groups.

Parameters	Group A (37 eyes)						Group B (42 eyes)					
	Univariate linear regression			Multivariate linear regression			Univariate linear regression			Multivariate linear regression		
	R^2^	*β* (95%CI)	*p*	*β* (95%CI)	*p*	*VIF*	R^2^	*β* (95%CI)	*p*	*β* (95%CI)	*p*	*VIF*
AL (mm)	0.030	−0.254 (−0.749, 0.242)	0.306	−0.154 (−0.567, 0.259)	0.453	1.099	0.006	−0.148 (−0.749, 0.452)	0.620	−0.345 (−1.001, 0.310)	0.292	1.469
ACD (mm)	0.032	−0.557 (−1.615, 0.501)	0.292	−0.171 (−1.284, 0.943)	0.757	1.751	0.006	0.384 (−1.232, 2.001)	0.633	1.046 (−1.212, 3.305)	0.354	2.403
LT (mm)	0.039	0.550 (−0.391, 1.492)	0.243	0.267 (−0.695, 1.229)	0.575	1.640	0.003	0.278 (−1.335, 1.890)	0.730	1.142 (−0.785, 3.068)	0.237	1.763
Lens tilt (°)	0.367	0.767 (0.421, 1.113)	<0.001	0.783 (0.441, 1.124)	<0.001	1.010	0.212	0.774 (0.297, 1.251)	0.002	0.759 (0.267, 1.250)	0.003	1.038
Lens decentration (mm)	0.043	−2.345 (−6.150, 1.459)	0.219	−2.277 (−5.502, 0.949)	0.160	1.123	0.027	2.218 (−2.024, 6.459)	0.297	2.312 (−1.638, 6.263)	0.243	1.046

AL, axial length; ACD, anterior chamber depth; LT, lens thickness. For Group A, the multiple linear regression yielded an R^2^ of 0.462 and an Adjusted R^2^ of 0.376. For Group B, the multiple linear regression resulted in an R^2^ of 0.279 and an Adjusted R^2^ of 0.179.

Both univariate and multivariate linear regression analyses indicated that IOL decentration in both groups was not significantly correlated with AL, ACD, LT, preoperative crystalline lens tilt, or decentration (all *p* > 0.05, Table 3). Moreover, no significant correlations between postoperative BCVA, postoperative astigmatism magnitude, and IOL tilt and decentration (all *p* > 0.05).

**Table 3:** Univariate and multivariate linear regression analyses of IOL decentration with preoperative parameters in both groups.

Parameters	Group A (37 eyes)						Group B (42 eyes)					
	Univariate linear regression			Multivariate linear regression			Univariate linear regression			Multivariate linear regression		
	R^2^	*β* (95%CI)	*p*	*β* (95%CI)	*p*	*VIF*	R^2^	*β* (95%CI)	*p*	*β* (95%CI)	*p*	*VIF*
AL (mm)	0.041	−0.019 (−0.051, 0.013)	0.231	−0.021 (−0.054, 0.013)	0.219	1.099	0.008	0.025 (−0.063, 0.112)	0.574	−0.032 (−0.137, 0.072)	0.533	1.469
ACD (mm)	0.005	−0.014 (−0.084, 0.055)	0.677	0.022 (−0.069, 0.112)	0.629	1.751	0.067	0.192 (−0.037, 0.420)	0.098	0.307 (−0.053, 0.667)	0.092	2.403
LT (mm)	0.040	0.037 (−0.025, 0.098)	0.233	0.059 (−0.019, 0.137)	0.133	1.640	0.002	−0.036 (−0.271, 0.199)	0.760	0.167 (−0.140, 0.474)	0.277	1.763
Lens tilt (°)	0.003	0.005 (−0.024, 0.033)	0.746	0.002 (−0.026, 0.030)	0.894	1.010	0.023	0.037 (−0.041, 0.114)	0.342	0.025 (−0.053, 0.104)	0.516	1.038
Lens decentration (mm)	0.042	0.150 (−0.097, 0.398)	0.226	0.209 (−0.053, 0.471)	0.114	1.123	0.045	0.418 (−0.195, 1.031)	0.176	0.356 (−0.274, 0.985)	0.259	1.046

AL, axial length; ACD, anterior chamber depth; LT, lens thickness. For Group A, the multiple linear regression yielded an R^2^ of 0.161 and an Adjusted R^2^ of 0.025. For Group B, the multiple linear regression resulted in an R^2^ of 0.140 and an Adjusted R^2^ of 0.020.

## Discussion

Building on our previous research, this study utilized a CNN with SS-OCT to compare the positioning of monofocal IOLs featuring plate-haptic and three-piece C-loop designs. The findings revealed that the three-piece C-loop design showed greater postoperative decentration than the plate-haptic design, although no significant difference in tilt was observed. The crystalline lens and IOL predominantly leaned toward the inferotemporal quadrant, with a notable positive correlation between their tilts.

An IOL usually displays a reasonable degree of tilt and decentration. The factors contributing to IOL tilt and decentration can be attributed primarily to three aspects (8, 20–22): (1) Patient factors: Research findings revealed that highly myopic eyes with AL ≥ 30 mm are prone to IOL decentration after surgery due to zonular laxity (23). Moreover, AL negatively correlates with IOL tilt (2, 24). Additionally, capsular bag contraction creates uneven circumferential traction forces on the IOL, further increasing the risk of IOL tilt and decentration (25). Furthermore, gravitational forces and zonular support change when patients transition from sitting to supine positions, potentially affecting IOL positioning. (2) IOL material and design: Table 4 offers a retrospective summary of previous studies, indicating that IOLs with various three-piece C-loop designs typically display more pronounced tilt and decentration values. In contrast, IOLs with open or full-plate haptic designs exhibit minimal tilt and decentration. On the other hand, hydrophobic IOLs, compared to hydrophilic IOLs, can reduce the risk of capsular bag contraction (21), thereby alleviating IOL tilt and decentration. Additionally, angulated haptic-optic junction position, orientation, and sharp-edge designs (26, 27), which may contribute to IOL decentration, negative dysphotopsia, and the incidence of posterior capsule opacification (PCO), further influence IOL stability. (3) Surgical factors: intraoperative posterior capsular rupture, zonular dehiscence, eccentric or irregular capsulorhexis (21), and vitreous prolapse could also increase the IOL tilt and decentration (28).

**Table 4:** IOL tilt and decentration with various haptic designs from previous studies.

Author	Year	IOL types (eyes)	Method	Result		
				Tilt (°)	Decentration (mm)	
Mutlu et al. (38)	2005	Single-piece C-loop: SA30AL (43 eyes) Three-piece C-loop: MA30BA (45 eyes)	Purkinje	SA30AL (2.70 ± 0.84) MA30BA (2.72 ± 0.55)	SA30AL (0.34 ± 0.08) MA30BA (0.39 ± 0.13)	
Hayashi et al. (39)	2005	Single-piece C-loop: SA30AL (30 eyes) Three-piece C-loop: MA60BM (30 eyes)	Scheimpflug	SA30AL (1.64 ± 1.00) MA60BM (1.67 ± 0.94)	SA30AL (0.24 ± 0.18) MA60BM (0.22 ± 0.13)	
Crnej et al. (40)	2011	Three-piece C-loop: MA60AC (15 eyes) Single-piece C-loop: SA60AT (15 eyes) Open-loop plate haptic: Akreos Adapt (30 eyes)	Purkinje	Akreos Adapt (Vertical 1.50 ± 1.10; Horizontal 2.90 ± 0.90) SA60AT (2.20 ± 7.20) MA60AC (5.30 ± 2.40)	Akreos Adapt (Vertical 0.40 ± 0.20; Horizontal 0.40 ± 0.20) SA60AT (0.40 ± 0.30) MA60AC (0.60 ± 0.80)	
Zhong et al. (41)	2016	Single-piece C-loop: ZCB00 (40 eyes) Three-piece C-loop: ZA9003 (40 eyes)	Scheimpflug	ZA9003 (1.01 ± 0.45) ZCB00 (1.06 ± 0.49)	ZA9003 (0.20 ± 0.17) ZCB00 (0.22 ± 0.17)	
Meng et al. (42)	2020	Single-piece C-loop: ZMB00 (59 eyes) Full plate haptic: 839MP (63 eyes)	OPD-Scan III	-	21.0 mm<AL ≤ 24.5 mm	839MP (0.14 ± 0.08, 30 eyes)
						ZMB00 (0.19 ± 0.11, 28 eyes)
					AL>24.5 mm	839MP (0.16 ± 0.10, 33 eyes)
						ZMB00 (0.41 ± 0.15, 31 eyes)
Xiao et al. (11)	2021	Open-loop single-piece C-loop: 920H (25 eyes) Single-piece C-loop: Softec HD (24 eyes) full plate haptic: 509 M (19 eyes)	CASIA2	509 M (4.96 ± 0.89) 920H (5.52 ± 1.74) Softec HD (6.28 ± 1.83)	509 M (0.12 ± 0.06) 920H (0.19 ± 0.12) Softec HD (0.22 ± 0.13)	

AL, axial length; −, no data reported; Vertical, IOL was positioned vertically; Horizontal, IOL was positioned horizontally.

Kimura et al. (10) measured crystalline lens tilt and decentration under natural pupil conditions in 41 eyes of cataract patients, reporting a tilt of 5.15 ° toward the inferotemporal direction relative to the corneal topographic axis and a decentration of 0.11 mm. Gu et al. (2) evaluated 56 subjects with SN60WF implantation under similar conditions, finding the lens and IOL tilt to be (4.90 ± 1.81)° and (4.75 ± 1.66)°, respectively, while the lens and IOL decentration both were (0.21 ± 0.02) mm. Preoperative crystalline lenses and postoperative IOLs were tilted toward the inferotemporal direction in the right and left eyes. Additionally, Kim et al. conducted an IOL tilt and decentration analysis 12 months post-surgery in 78 subjects, yielding results of (4.20 ± 1.70)° for tilt and (0.24 ± 0.15) mm for decentration (29). Although all of these studies used SS-OCT as the imaging modality, there are both similarities and discrepancies in the findings. These variations may be attributed to differences in the baseline characteristics of the study populations, sample sizes, IOL types, and the analysis algorithms implemented. In the present study, both groups of crystalline lenses and IOLs generally tilted toward the inferotemporal direction, exhibiting mirror symmetry between the eyes, which aligns with previous research results (24, 30).

Unlike the IOL types examined in previous studies (Table 4), this research includes a single-piece plate-haptic IOL and a three-piece IOL (31, 32). The postoperative decentration measured in Group A (0.16 ± 0.08 mm) was significantly smaller than in Group B (0.42 ± 0.28 mm). The disparity may be attributed to differences in IOL design and size. The 709 M has four distinct points contacting the capsular bag with an approximate total contact length of 12.53 mm. Conversely, the PY-60 AD incorporates a two-point contact configuration, with an estimated total contact length of 12.50 mm. The integrity of IOL positioning can be compromised by factors such as postoperative anterior capsule contraction, capsulotomy size, and centration, all of which contribute to an asymmetrical distribution of forces on the IOL optic. The PY-60 AD’s two-point contact design is hypothesized to be more susceptible to these asymmetrical forces than the 709 M’s four-point design, potentially leading to greater decentration.

In this study, IOL tilt showed a positive correlation with crystalline lens tilt in both groups; however, IOL decentration did not correlate significantly with preoperative ocular parameters. Gu et al. (2) used CASIA2 to measure tilt and decentration in 56 eyes (with AL ranging from 21.38 to 32.27 mm) that received a single-piece C-loop SN60WF IOL during surgery. In a multiple linear regression analysis, they found that postoperative IOL tilt was significantly related to preoperative lens tilt (*β* = 0.565, *p* < 0.001), AL (*β* = −0.173, *p* = 0.033), and LT (*β* = −1.009, *p* = 0.011). Furthermore, IOL decentration correlated with lens decentration (*β* = 0.620, *p* < 0.001) and AL (*β* = 0.041, *p* < 0.001). The differences observed between their results and those of this study may be partly attributable to variations in measurement equipment, the characteristics of the study populations (with AL ranging from 21.00 to 26.0 mm), the duration of follow-up, and the reference axes (this study utilized the corneal vertex as the reference, while CASIA2 relied on the corneal topography axis (33)). Nevertheless, there is a consistent positive correlation between the crystalline lens tilt and IOL tilt across these studies.

While a certain degree of IOL tilt (up to 2–3°) and decentration (0.2–0.3 mm) are often clinically unnoticed with many IOL designs, it is important to recognize that these values are averages, and individual patient experiences may vary (9, 34). Optical bench analyses further demonstrate that decentration can result in a significant reduction in modulation transfer function values and an increase in wavefront aberration errors (35). Research by Ruiz-Alcocer et al., using an optical bench, has shown that IOL tilt and decentration can diminish through-focus optical quality, particularly with premium IOLs and post-LASIK corneas (3–5). The lack of significant correlation between postoperative astigmatism magnitude, BCVA, and IOL tilt and decentration in this study is primarily attributable to the relatively narrow range of tilt and decentration observed and to the use of a monofocal lens.

This study presents several limitations: Firstly, as a retrospective study, the intraocular higher-order aberrations (HOAs) were not assessed. Secondly, the follow-up period in the current study is relatively short, and the results from the first postoperative month mainly reflect the immediate postoperative force. They may not be directly used to infer long-term stability differences. Also, the manual capsulorhexis quality, especially the capsulorhexis size, circularity, centration, and the IOL material properties, IOL weight, could also affect the IOL stability in the long term (36, 37). Therefore, future prospective investigations with a larger sample size, longer follow-up, and more capsulorhexis metrics, visual quality parameters (including HOAs) will offer deeper insights into the long-term stability and visual impact of IOL positioning. However, we still believe that the current study will provide valuable information on premium IOLs and Toric IOLs with similar haptic designs for clinical reference in the short term (4, 5).

In conclusion, this research underscores that plate-haptic design monofocal IOLs exhibit less postoperative decentration compared to C-loop design IOLs. Additionally, a significant positive correlation was observed between the crystalline lens and the IOL’s spatial position. Therefore, clinicians should consider the preoperative position of the crystalline lens and the potential differences in IOL decentration across different haptic designs during IOL selection.

## Data Availability

The original contributions presented in the study are included in the article/supplementary material, further inquiries can be directed to the corresponding author.
